# Child maltreatment, attachment and psychopathology: A case report

**DOI:** 10.1192/j.eurpsy.2021.1666

**Published:** 2021-08-13

**Authors:** M.D.C. Molina Lietor, I. Cuevas, M. Blanco Prieto

**Affiliations:** Psiquiatría, Hospital Universitario Príncipe de Asturias, Alcalá de Henares, Spain

**Keywords:** child maltreatment, reactive attachment disorder, adoption

## Abstract

**Introduction:**

The exposure to child maltreatment increases the lifetime risk for many psychopathological symptoms: depression, anxiety disorders, bipolar disorder, schizophrenia, post-traumatic stress disorder, personality disorder and dissociation. Besides, adopted children, especially those with a history of institutional living before adoption, are at greater risk for a range of developmental, behavioral and attachment concerns. The case report is of a 17-year-old male, with reactive attachment disorder (RAD). He suffered child maltreatment in his family of origin before the international adoption.

**Objectives:**

The aim of this study is to present a case-report illustrating the relationship between chil maltreatment, adopted children and the reactive attachment disorder.

**Methods:**

A bibliographic search was performed about reactive attachment disorder. Information regarding the clinical case was obtained by consulting the patient’s file.

**Results:**

A 17-years-old male who was adopted at age of 9 from Spain. According to reports from the orphanage, the patient suffered severe maltreatment by his family of origin, with scars on his back. The patient presents impulse control disorder, with verbal and physical heteroaggressiveness in situations of frustration, hunger and sleep. He stopped attending the institute at the age of 12, with marked isolation and reversal of the sleep-wake cycle. His treatment plan are partial hospitalization, psychotherapy and pharmacotherapy.

**Conclusions:**

Both child maltreatment and adoption are risk factors for the presence of psychopathology during the lifetime. Especially during the pre-adoption process and the first years after adoption, both the family and the child should be able to use specialized Mental Health services.

**Disclosure:**

No significant relationships.

